# A Video-Based Reflective Design to Prepare First Year Pharmacy Students for Their First Objective Structured Clinical Examination (OSCE)

**DOI:** 10.3390/healthcare10020280

**Published:** 2022-01-31

**Authors:** Vivienne Mak, Daniel Malone, Nilushi Karunaratne, Wendy Yao, Lauren Randell, Thao Vu

**Affiliations:** Pharmacy and Pharmaceutical Sciences Education, Faculty of Pharmacy and Pharmaceutical Sciences, Monash University, Parkville, VIC 3052, Australia; Dan.Malone@monash.edu (D.M.); nilushi.karunaratne@monash.edu (N.K.); wyao0001@student.monash.edu (W.Y.); lsran2@student.monash.edu (L.R.); Thao.Vu1@monash.edu (T.V.)

**Keywords:** e-learning, healthcare education, OSCE, video, reflective practice, feedback

## Abstract

We explored the use of a video-based reflective design in preparing first-year pharmacy students for their Objective Structured Clinical Examination (OSCE) in Victoria, Australia. This involved pre-workshop activities (a recording of themselves simulating the pharmacist responding to a simple primary care problem, written reflection, review of the OSCE video examples and pre-workshop survey); workshop activities (peer feedback on videos) and post-workshop activities (summative MCQ quiz and post-workshop survey). These activities took place three weeks before their OSCE. A mixed-method study design was employed with quantitative and qualitative analyses of the surveys and a focus group. A total of 137 students (77.4%) completed the pre- and post-workshop surveys, and ten students participated in the focus group. More student participants (54%) reported feeling prepared for the OSCE post-workshop than pre-workshop (13%). The majority (92%) agreed that filming, watching and reflecting on their video allowed them to learn and improve on their skills for the OSCE. The regression analysis found that video recording submissions and written reflections correlated positively with student OSCE performances, and the video-based reflective design learning experience was perceived to be beneficial in multiple ways. Thematic analysis of the focus group data revealed that students acquired metacognitive skills through the self-assessment of their video recordings, developed an awareness of their learning and were able to identify learning strategies to prepare for their first OSCE. Fostering students’ feedback literacy could be considered in future educational designs.

## 1. Introduction

Video-based reflective design incorporates the use of technology, in this instance, video recordings, as a medium for self-reflection and feedback. The observation of self-performance through video recordings allows for a review of how one’s self is portrayed and to facilitate self-directed learning [1]. Multiple studies have outlined the benefits of improving self-evaluation and communication skills through the review of video recordings [2,3,4,5,6]. It is suggested that, when weaknesses are identified in one’s own performance through self-evaluation, one is more likely to be motivated to consider steps to improve [7]. There is an increasing uptake in the utilisation of video recording and reviewing activities within teaching, nursing, medical and pharmacy courses globally. It is proposed that communication skills will improve in pharmacy students through the feedback and reflection of simulated role play recordings [8]. One study observed an increase in self-perception following a video review of a counselling activity conducted by first-year pharmacy students and supported the inclusion of an activity that reinforced self-evaluation [5]. The concept of metacognition, in which self-evaluation is an important component, continues to be explored as an important skill for pharmacy students to develop over time [9]. Metacognition has been defined in various ways but is generally considered to be the process of reflecting on and evaluating one’s own learning [10]. The metacognitive process of self-regulation and reflection on knowledge and skills is important for health professional students for self-improvement [11].

In this current study, we implemented a video-based reflective design to prepare students for their first Objective Structured Clinical Examination (OSCE). OSCEs have been established as an effective means of evaluating pharmacy students’ performances in a simulated environment [12,13]. OSCEs have been used as summative assessment tasks to assess problem-solving and communication at the Monash University Faculty of Pharmacy and Pharmaceutical Sciences since 2011 [14]. Preparing healthcare students in terms of developing their problem-solving and communication skills can be done in numerous ways. These include, but are not limited to, the use of online virtual tools, video exemplars, teaching or mock OSCEs, peer assessment and feedback [15,16,17,18]. On the other hand, preparing students for OSCEs using teaching or mock OSCEs and instructor feedback can be challenging due to resource constraints such as time barriers, additional staff workload and costs [19]. Generally, students with previous OSCE experiences, whether through an actual or mock OSCE, have experienced reduced anxiety and improved performances, although the implementation of mock OSCEs as described is limited by the associated costs [20].

Thus, self-directed learning and preparation for OSCEs by students themselves is important. The study strategies identified in the literature that students themselves use to prepare for OSCEs include practicing with other students, rehearsing routines, physical examination courses (practical classes), class notes/logs and skills labs [20,21]. Nevertheless, students found resources for gathering knowledge such as lectures or textbooks less helpful and spent less time studying as compared to multiple-choice question (MCQ) tests [20]. Many OSCE preparation techniques have been reviewed to help students achieve the optimal performance in OSCEs. These include various strategies reported that aim to improve metacognition in students as part of the OSCE preparation process. Teaching reflective skills and encouraging students to voluntarily practise and reflect on their clinical skills were shown to improve OSCE scores in medical students [22]. High achievements in pharmacy students have been linked to various metacognitive processes, including self-efficacy and self-evaluation [23]. A peer-led mock OSCE approach is a strategy identified to reduce the costliness of OSCE preparation whilst retaining the benefit of the mock OSCE experience [24,25]. However, peer-led mock OSCEs do not address any deficiency in students’ self-reflective capacities [25,26,27,28] that pharmacists may carry throughout their careers [29]. Therefore, if metacognitive skills can be incorporated as part of the pharmacy curriculum, this may lead to the better preparation of students for high stakes assessment tasks such as OSCEs.

The purpose of this study was to explore the use of a structured video-based reflective design and its influence in preparing first-year pharmacy students for their first OSCE. To our knowledge, there is limited literature linking the impact of a structured video-based reflective design utilizing students’ own video recordings, self-reflection of those video recordings and further reflection upon receiving peer feedback on those video recordings within Australian pharmacy programs on OSCE performances in pharmacy students.

The most common undergraduate pharmacy degree that is offered in Australia is the four-year Bachelor of Pharmacy (BPharm). At the Faculty of Pharmacy and Pharmaceutical Sciences, a new pharmacy program was developed that had its first cohort of year 1 students in 2017 [30]. One of the major distinguishing features of this new program is the whole degree flipped classroom delivery that includes self-directed learning, interactive lectures, weekly small group workshops and a skills coaching program that is focused on enhancing reflective practice and metacognitive skills. The new pharmacy program places an emphasis on skill development, including oral communication, empathy and problem-solving skills. Students enter the pharmacy course without the experience of a skills-based performance exam such as the OSCEs. Therefore, providing guidance on how to undertake, and perform well in, their first-year OSCE is essential.

A structured video-based reflective design to prepare students for their first-year OSCE was introduced with a series of preparatory activities. These activities included providing students with tasks that are easy to complete in their own time, with the aim to equip them with OSCE preparation strategies that they can retain throughout their degree. This study provides insight into how health professional schools can prepare students for their first OSCE utilising a structured video-based reflective design.

The research questions for this study were:

(1) How did the video-based reflective design prepare students for first-year OSCEs as perceived by the students?
(2) What is the relationship between student engagement in the video-based reflective design activities and their OSCE performance?

## 2. Materials and Methods

### 2.1. Video-Based Reflective Design

We designed a video-based reflective design to prepare students for their first OSCE. This included pre-workshop tasks, workshop activities and a post-workshop task.

**Pre-workshop:** Students completed a summative assessment worth 2% of the course mark. There were two components to this summative assessment: (1) video recording submission and (2) written reflection submission. For the video recording, students had to film themselves performing a short *role play interaction* (5 min maximum) simulating a pharmacist responding to one simple primary care problem with a mock patient. The eight primary care topics included diarrhoea, threadworm, constipation, headache, tinea, hay fever, reflux and the common cold. Students were provided with one case topic and the corresponding script. Each student selected their own partner as the mock patient to complete the task. For the *written reflection*, students were asked to review their recordings and, based on their videos, respond to three structured points: reflect on their accomplishments, identify areas for improvement and identify strategies to improve. The 2% of the course mark was awarded as long as the student submitted both the video recording and written reflection (*summative pre-workshop video and reflection submission*). The content of the video recording and written reflection was not assessed. Although not compulsory, students were encouraged to self-evaluate the video using the *OSCE communication rubric* provided to them. Students then attended two face-to-face 50-min large class interactive lectures that included *watching a previous student’s OSCE video as examples* and information on the OSCE process. Whilst watching the video examples, students were asked to formatively assess the performances in the videos using the OSCE communication rubric. Prior to attending the workshop, the students were invited to complete a 1-item survey asking how prepared they were for the OSCE (*pre-workshop survey*).

**Workshop:** Three days after the completion of the pre-workshop tasks, students attended a two-hour workshop (*workshop attendance*). At the commencement of the workshop, the workshop facilitator, who was also a practicing pharmacist, played two exemplar videos to the students, with one video more ideal than the other. Students were then asked to provide feedback on the exemplar videos using the same structure as their pre-workshop written reflections and with using the OSCE communication rubric. The workshop facilitator then provided the students with feedback on the quality of their feedback on the exemplar videos. Students were then divided into small groups of 5 to 6 students per group and were told to use the same feedback structure in the following workshop task. Each student then played their own recording to their peers, verbally shared their written reflections from the pre-workshop and received peer feedback on their video. Each student repeated this process until all the students played their own recordings.

**Post-workshop:** After completion of the workshop, students were invited to complete a 4-item survey evaluating how helpful the workshop was and how prepared they felt for the OSCEs (*post-workshop survey*). Students then had one week to complete a summative multiple-choice question (MCQ) quiz on the eight primary care topics. This was a content knowledge quiz with 20 MCQs with a 60-min time limit. Students could attempt this twice, with the final attempt counting towards their course mark and worth 8% (*summative post-workshop MCQ quiz mark*).

### 2.2. Study Design and Participants

This was a mixed-method study design where we triangulated both quantitative with qualitative analyses to answer our research questions. Participants in this study were first-year pharmacy students at Monash University Australia in 2017. A total of 177 students were invited to complete the pre- and post-workshop surveys. The same 177 students were invited to participate in the focus group. The study was approved by the Monash University Human Research Ethics Committee. As part of the standard quality improvement processes for the pharmacy degree and consistent with the Student Privacy Collection Statement, student performance data were collected and deidentified.

#### 2.2.1. Pre- and Post-Workshop Survey

Students were invited to complete a 1-item perception survey about OSCE preparedness before the workshop commenced. Following the workshop, students were invited to complete a 4-item perception survey about the usefulness of the workshop activities. Specifically, these items were used to determine whether students self-recording and reflecting on their performance helped students learn and improve their skills for the OSCEs and, secondly, whether reviewing recordings of other students helped students learn and improve their skills for the OSCEs. Student perceptions on OSCE preparedness were also recorded at the completion of the workshop. Data were collected using a self-administered online survey based on the objectives of the research. This survey was conducted on the learning management system Moodle. Data were analysed using descriptive analysis (percentage agreement with each survey item).

Three regression analyses were performed to determine whether correlations existed between the students’ OSCE performances and various video-based reflective design activities.

These analyses were completed with the three dependent variables:

(1) *OSCE communication mark*—graded using an OSCE communication rubric and assesses skills;
(2) *OSCE analytical checklist mark*—graded using a checklist that assesses clinical points;
(3) *Overall OSCE mark*—total mark combining both communication and analytical checklist marks.

For each analysis, the independent variables were *summative pre-workshop video and reflection submission, workshop attendance* and *summative post-workshop MCQ quiz mark* (as described in Section 2.1). Statistical significance was defined when *p* < 0.05. All statistical analyses were conducted using SPSS Version 23 (IBM Corporation, Armonk, NY, USA).

#### 2.2.2. Focus Group

Ten students (seven female, three male) who had completed both the pre- and post-workshop surveys and their first-year OSCE volunteered to participate in an hour-long focus group facilitated by two researchers (T.V. and N.K.). These two researchers were not part of the students’ direct teaching staff. As the students completed the same activity in their first year, their shared experience was conducive to facilitating an interactive reflection of their first-year OSCE preparation, which is essential for using the focus group data collection method [31]. To minimise bias due to potential pre-existing relationships among the student participants, a number of strategies were employed. Firstly, the students were briefed on the aims and the format of the focus group and were invited to participate in and cocreate a safe and nonjudgmental environment to share individual ideas about their experiences. Secondly, to ensure all students had the opportunity to share their personal perspectives, the students were asked to complete a list of individual brainstorming tasks before taking turns responding to open-ended questions prepared in advance by the researcher facilitators. Students were regularly prompted with “How?” and “Why?” questions to explain their own answers. Thirdly, students were frequently encouraged during the focus group to agree, disagree and elaborate on each other’s ideas.

The focus group was audio-recorded with the student participants’ consent. The audio recording was transcribed and deidentified. Focus group data as password-protected files were only accessible by the researchers. Two researchers (T.V. and N.K.) analysed the data together using thematic analysis [32] to answer the research question: How did the video-based reflective design prepare students for the OSCEs? The data was categorised according to the main components of the activity: (1) filming, watching and reflecting on their own role play video using a structured approach; (2) watching and evaluating example videos of student OSCE performances and (3) watching and evaluating example videos of student OSCE performances. Following this structure, a mutually agreed codebook was developed. Given the relatively small amount of qualitative data, the coding was conducted using a shared Google Doc, Google Sheet and extensive discussions between the two coders. The codes and the individually induced themes were thoroughly discussed, moderated and triangulated by the two coders to reach a consensus on the final themes [31,33]. The codes from the focus group data analysis are explained in Section 3.1.1, Section 3.1.2 and Section 3.1.3. Two emerging themes related to students acquiring metacognitive knowledge through self-reflection and feedback literacy are discussed in Section 4.

## 3. Results

A total of 137 students completed the pre- and post-workshop surveys (77.4%). Student performance and engagement data were collected from all 177 students. A total of ten students participated in the focus group.

### 3.1. How Did the Video-Based Reflective Design Prepare Students for the First Year OSCEs as Perceived by the Students?

This section presents the research findings in response to the first research question. The data from both the pre- and post-workshop survey results and focus group showed that the student participants felt more prepared for the first-year OSCE after the workshop and reportedly benefited from all the three components of the video-based design: the video recording, watching and reflecting on their own or their peers’ role play videos (Table 1). When students were engaged in the activity and adopted a structured approach to reflect on their own video and their peers’ videos, the learning experience was perceived to be beneficial in multiple ways that will be explained below. These research findings shed light on two interrelated emerging themes (discussed in Section 4) about the acquisition of metacognitive knowledge through self-reflection and feedback literacy that are essential to this activity.

Overall, a greater proportion of student participants (54%) who completed the survey reported feeling prepared for the OSCEs after the workshop compared to only 13% before the workshop.

To shed light on what specifically students found useful about these activities in helping them prepare for the OSCE, the focus group data showed that all the activities contributed to enhancing students’ awareness of their own areas for improvement and their ability to reflect and provide feedback on an OSCE performance, which helped them feel more able to self-regulate their learning and practice of their skills.

#### 3.1.1. Filming, Watching and Reflecting on Their Own Role Play Video Using a Structured Approach

In this activity, students filmed, watched and reflected on their own role play video with their own selected mock patient using the provided *OSCE communication rubric* and a structured approach. There was a strong consensus among the focus group students that this activity was “*very helpful*” (Student 5) for several reasons.

Most importantly, students reported that they became more **self-aware of the areas to improve in communication skills** and that they were able to **draw on their gained self-awareness to regulate their own practice for the OSCE**. For example, some students explained that they were able to observe their own performances objectively and become cognizant of specific behaviours that might affect their communication:

*“On the video **I saw I was fidgeting a lot; moving around in my chair**. So that was a good way to find out how to improve yourself.”*

(Student 7)

*“I also thought videoing ourselves was really valuable… I watched mine many times, I was like, That’s another thing I can pick up, that’s another thing I shouldn’t be doing.”*

(Student 2)

*“I felt that* *was pretty good because you could actually see the mistakes you did throughout the actual video.”*

(Student 5)

A student reportedly utilised this new knowledge of their own performance to inform their preparation for the OSCE:

*“…we were able to come to terms with where we were at the moment and we still had a few weeks until the OSCE to improve upon that for the OSCE.”*

(Student 3)

In addition, the students also found filming their role play videos helpful because the activity **prompted them to practice and learn through practice**. For instance, one student said that, by working on the role play counselling video, they practised “*how to actually communicate to each other, how to set a structure as to how we go about collecting information and giving out information about medication*”. (Student 2). Another student contended that, by working with other students to film the video, they could “*pick up*” good strategies from their peers and “*incorporate*” those into their own counselling (Student 8).

Furthermore, by reflecting on their own video using the provided structured approach with the *OSCE communication rubric*, the student reported the **benefit of interacting with the rubric to be used in the exam** and became more aware of “*what we’re looking for*” in terms of evaluating their own performance and “*what we should aim towards*” in terms of preparing for their future OSCE performances (Student 9). The gained awareness was then used to “*guide*” their own practices for the OSCE (Student 9).

#### 3.1.2. Watching and Evaluating Example Videos of Student OSCE Performances

In this activity, students attended two interactive lectures where they were shown examples of past videos of student performances in the OSCEs and were instructed to use the OSCE communication rubric to evaluate the example performances. There was a strong agreement in the group that the knowledge gained from this activity “*demystified the whole OSCE process*” and provided them with a perspective of what worked and what could go wrong in an OSCE performance.

The students reflected that, because they had no prior experience with OSCEs, the opportunity to “*go through the exemplars… what not to do and what to do*” was “*really invaluable*” (Student 4). Without this instruction, the students felt that they would have felt “*blank*” during in the actual exam (Student 6) and their performances could have been negatively affected because their first OSCE could be “*an overwhelming experience*” (Student 5).

The perspectives gained from various video examples were also reportedly helpful for students’ self-reflection and sense of confidence. For example, one student commented:

*“My favorite part was when they used to show us videos of a really bad OSCE and really good OSCE. They seem really obvious and people laugh at them, as if you would do an OSCE that bad, but **sometimes you can see something in yourself that you’re doing in that video**, or it’s a bit of confidence. At least I’m not doing that. They were really good, those videos.”*

(Student 3)

Students suggested that the activity could be improved with the use of an audience engagement tool like Poll Everywhere to encourage more students to provide feedback and/or to ask for clarification. Another recommendation that some students shared was to make the example videos used in the lecture accessible afterwards for reviewing.

#### 3.1.3. Reviewing and Providing Feedback on Peer’s Role Play Videos

In this activity, students participated in a workshop where they worked in small groups and provided peer feedback on one another’s videos that they had submitted in the previous activity. The most common view among the focus group participants was that, **when students were engaged** in sharing their own videos and providing structured feedback, they **benefited from gaining more awareness of what they could improve and what they could learn from their peers**. The students believed that this practice was helpful for both the **short-term OSCE preparation** and their **development of professionalism in the long run**. However, a few students found this activity not as helpful when they or their peers were hesitant to participate or when they perceived the provided feedback from peers was too general.

What the focus group students found most helpful about the peer feedback activity was **the various perspectives about how they could improve their performance**. For instance, one student reflected positively on the variety and objectivity of the feedback received from the students randomly allocated to their workshop group, which could be different from the feedback they usually received from their friends:

*“…we were a group of six, so you **get more opinions**… my friends focus more on the content, but…they [other students in the workshop group] focus more on the other aspects of communication, like non-verbal, what you could do to improve your performance… I personally felt it was good because I was given **different opinions from six different people that I don’t really talk to… Their objective views were really nice** because when I ask my friends about it [my OSCE video] they’re like, Oh, that sounds great. When you ask someone else they’re more like, You need to fix this, you need to fix that, and I was like, Okay, I shall do that next time round. So personally I liked it.”*

(Student 9)

Similarly, another student acknowledged that the peer feedback helped them **understand their strengths and weaknesses better** so that they could prepare for their OSCE performance:

*“…in my opinion, if you kept the video to yourself you wouldn’t be able to understand what you did wrong. Whereas, my friend [student in the workshop group] told me what **I needed to work on as well as what I needed to fix up**. That then gave me **a good understanding of what I actually did well.**”*

(Student 7)

Nevertheless, a minority of students held a different view that peer feedback group activity **might not be helpful when the feedback they received from their peers was not specific enough**. One student contended that “*showing that [the video] to someone else and having them say, Ah, you didn’t really do a great job, is not helpful for me*” (Student 3). In this case, the workshop group appeared not to follow the structured approach using the OSCE communication rubric to provide feedback as instructed. Another hindrance to the usefulness of the workshop activity was that the students **declined to show their own videos** to the group or were **reluctant to provide peer feedback**. The same student who did not find peer feedback helpful admitted refusing to show their own video because they “*felt like they had a long way to go” and “having to show that to a peer, I just think it’s embarrassing*” (Student 3). Another student observed that their group was “*reluctant to talk*” and “*reluctant to give feedback*” (Student 9). The obstacle to really benefiting from this activity seemed to relate to **the sense of safety** students have in terms of receiving and providing feedback in a group setting and, potentially, their **level of feedback literacy**.

Whilst exposing one’s own potential weaknesses and receiving feedback can be “*daunting*” to students, the skill was believed to benefit the students’ **long-term growth in professionalism**. One student reflected that it was important to develop this skill as part of their professionalism:

*“Initially it’s very daunting and to present [one’s own video] in front of other people is even more daunting. I think from a professional standpoint, it’s a skill that you have to develop because you’re going to get feedback from people you do not want to get feedback from anyways.”*

(Student 5)

Moreover, some students acknowledged that providing peer feedback and observing others’ performances was a useful learning experience in which they **transferred their feedback of other performances to what they could improve in their own performance**:

*“I was able to see their OSCE videos as well, so I was able to see… what they did in their video and I was like, Oh, okay, so I need to do that… Or how to phrase some words when counseling patients, so that was nice.”*

(Student 9)

*“…by looking at other people’s videos we could see, Okay, they did that, I want to do that. Or They didn’t do that, I would have put that in my video.”*

(Student 10)

In summary, according to most of the student participants in both the survey and focus groups, the video-based reflective design was useful in preparing students for their first OSCE. When students were engaged in the activity and adopted the structured approach with the OSCE communication rubric to reflect on their own video and others’ videos, the learning experience was perceived to be beneficial in multiple ways. Filming the video prompted students to practise what they had learnt during the course. Reflecting on their own performance and others’ using the OSCE communication rubric helped enhance the awareness of their own and others’ strengths and gaps in skills that informed their regulation of practising the skills to prepare for the OSCE. Providing peer feedback helped students learn from each other and potentially nurtured the necessary mindset and skill to receive feedback as part of their professionalism. From the focus group data, what appeared to be important influencing factors for the students to gain these benefits could be their sense of safety and ability in dealing with the discomfort of letting others see and evaluate their (perceived) “*imperfect*” performances, as well as their feedback literacy, particularly the ability to provide effective specific feedback to their peers.

### 3.2. What Is the Relationship between Student Engagement in the Video-Based Reflective Design Activities and Their OSCE Performance?

Multiple linear regression analyses found significant effects of the teaching activities on the *overall OSCE mark* (F_3136_ = 4.110, *p* < 0.05), *OSCE communication mark* (F_3136_ = 4.024, *p* < 0.05) and the *OSCE analytical checklist mark* (F_3136_ = 3.312, *p* < 0.05). The *summative pre-workshop video and reflection submission* were the only significant predictors of the *overall OSCE mark* (*p* = 0.001), *OSCE communication mark* (*p* = 0.001) and *OSCE analytical checklist mark* (*p* = 0.007) (Table 2). This indicates that students that created a video and submitted a reflection performed better in the OSCEs, both from the perspective of communication and achieved scores on the OSCE analytical checklist, than students that did not.

## 4. Discussion

This study offers several findings on the use of video-based reflective designs in preparing students for their first Objective Structured Clinical Examinations (OSCEs) in pharmacy education. It is an innovative structured video-based reflective design incorporating video recordings, self-assessment and exemplar videos with peer feedback, and this study linked the impact of this design on pharmacy students’ first OSCE performance. This study found that more students perceived that they felt more prepared for their OSCE after the workshop compared to pre-workshop. It was found that the students’ perceptions of their preparedness for the OSCEs drastically improved from 13 to 54% after participating in the workshop. Although 39% of student participants did not feel either way, most students (92%) agreed or strongly agreed that the process of filming, watching and reflecting on their role play videos allowed them to learn and improve on their skills for their OSCE. These findings are consistent with a previous study, albeit it was conducted on fourth-year pharmacy students, where video reviews of their simulated role plays were shown to be beneficial in facilitating awareness of their nonverbal communication [4]. This was further substantiated by the qualitative data in our study demonstrating that the video-based reflective design activities stimulated students’ ability to identify their strengths and areas for improvement in their communication skills to consider the learning strategies for further improvement. They found the feedback received and given to their peers useful for their own learning and preparation for their OSCE. Importantly, our findings demonstrated better performances in the OSCEs by students who undertook the video-based reflective design activities, as evidenced by a regression analysis. A similar outcome was observed in a study with nursing students that found students were more satisfied with their learning and noticed a significant positive impact on summative grades for students who participated in the video-based self-assessment [3].

Our study provided empirical findings around the usefulness of the video-based reflective design as part of the wider programmatic approach in developing metacognitive skills amongst pharmacy students. Metacognition helps the individual be more aware of and control their thinking processes. Classically described metacognition frameworks involve two components of metacognition: knowledge of cognition (metacognitive knowledge) and regulation of cognition (metacognitive regulation). This study integrated metacognitive development into the undergraduate curriculum through a video-based reflective design to prepare first-year pharmacy students for their first OSCE.

The students acquired metacognitive knowledge through self-reflection, especially among their peers in a classroom setting, providing learners the opportunity to develop these soft skills through the self-identification of gaps to be filled. The learning strategies of peer assessment and self-reflection have been demonstrated to quicken the process of the development of metacognitive skills among learners [34,35]. This is because a peer assessment often leverages on social pressure associated with learners not wanting to lose face in front of their peers. Learners are motivated to focus on the processes of self-reflection and self-regulation to avoid the embarrassment of having their colleagues openly identify the probable gaps in their learning among their peers [31]. Students were further able to identify the strengths and weaknesses in relation to their OSCE preparations when working with peers. This provided students with more robust knowledge and more accurate self-assessments of their abilities. Furthermore, engaging students in discussions around their self-recorded videos allowed students to compare their own performances against their peers, as well as reflect on improving their own skills. Students were also able to practice providing and receiving constructive feedback, which is an important quality that is necessary for ongoing development [36].

The video-based reflective design learning activity also created opportunities for learners to regulate their acquired metacognitive knowledge. Students reviewed their performance of the task and understood how the performance would be assessed. The activity enabled students to use the OSCE communication rubric to guide them toward the goals from practice and provided guidance on how to achieve these goals. Through this reflective practice of evaluation, metacognitive knowledge is built and refined, which implicates a feedback loop where new and better knowledge is used for the regulation of cognition, for example, resulting in better planning in the future.

In this paper, we explored how a video-based reflective design prepared students for their first OSCE. We found further evidence on how incorporating feedback literacy in educational design as part of the video-based reflective design could benefit student learning. Feedback could be understood as “*a process through which learners make sense of information from various sources and use it to enhance their work or learning strategies*” [37] (p. 1315). Students’ feedback literacy, which is fundamental to their learning and in their future work as a pharmacist, consists of four main dimensions: “*appreciating feedback; making judgments; managing affect; and taking action*” [37] (p. 1316). Our study showed that students’ learning was enhanced through the process of providing and receiving feedback, and they appreciated this ability as part of their long-term development in professionalism. The two activities, peer feedback and analysing video exemplars, in our design, were recognised as activities that facilitated their development in feedback literacy. We also found that students’ sense of safety and comfort in their peer group impacted their willingness to share their imperfect video recordings. Additionally, their perceived skill to provide actionable feedback influenced the quality and outcome of the learning experience. Previous literature has also described how a student is less likely to participate in providing peer feedback due to a lack of confidence in the task [38]. Therefore, we highly recommend including learning materials and activities to help students develop a positive mindset about receiving and giving feedback and the ability to “manage affect” feedback, as well as the skill to provide effective feedback at the outset of these activities [37] (p. 1316).

The results from the multiple regression analysis showed that there was no correlation between the *summative post-workshop MCQ quiz mark* and student performance in any aspects of the OSCE. It may have been expected to see a correlation between the *summative post-workshop MCQ quiz mark* and the *OSCE analytical checklist mark*, considering both were summative assessments based on students’ content knowledge. However, the *MCQ quiz* tested the ability of students to remember and understand material related to the OSCEs, as opposed to the *OSCE analytical checklist*, which was designed to assess the ability of students to analyse information in, and apply knowledge to, a clinical case. It has been previously shown that pharmacy students perform differently on assessments that focus on recall compared with the application or analysis of information [39,40].

Despite there being no correlation between *workshop attendance* having an impact on the *overall OSCE mark*, the findings of the focus group discussion suggested otherwise. The majority of the focus group students expressed that the workshop activity provided multiple learning opportunities not only from watching other student videos but also from receiving and providing constructive feedback. This entire process allowed each student to critique their own performance and identify areas of improvement so as to assist in the preparation for their first OSCE. The results of the multiple regression analysis regarding attendance may be explained by the minority of focus group students who considered the workshop activity as unhelpful. Students found the workshop unhelpful when other students were unwilling to share their own videos or received nonspecific feedback with no indication of how to improve their performance. Thus, it is important for educators to create a safe environment during the learning activity so that students feel safe to share their self-recorded videos and feel confident in providing and receiving feedback.

A strength of this study was the focus on one approach, the video-based reflective design as part of a programmatic approach to developing the core skills and knowledge for the OSCE. However, it is important to acknowledge that skill development can be influenced by a number of factors, including engagement in other activities within the program, workplace experiences and prior exposure to patient care activities. Future research should evaluate the program’s instructional design model as a whole in preparing students for the OSCE. A limitation of this research was that there was no guarantee that the peer feedback provided was constructive or helpful to students, as the quality of the feedback was not assessed. Nevertheless, there was an instructor present, but this instructor may not have provided oversight of every student. Additionally, the focus group consisted of 10 students in the cohort, and thus, the results may not be generalisable to the majority of students.

## 5. Conclusions

The video-based reflective design used in this study was found to be useful in the development of metacognitive skills, with video recordings and reflective tasks positively correlating with the OSCE performance outcomes. The video-based reflective practice helped enhance students’ awareness of their learning and stimulated them to consider various learning strategies according to their own learning needs to prepare for their first OSCE. Educators may also consider students’ feedback literacy in their educational design to further encourage student confidence in giving peer feedback so that a positive overall learning experience can be achieved.

## Figures and Tables

**Table 1:** Pre- and post-workshop survey results (*n* = 137).

Survey	Item	Strongly Disagree/Disagree	Neutral	Strongly Agree/Agree
Pre- workshop	I currently feel prepared for the OSCEs	36%	51%	13%
Post- workshop	After the workshop, I feel prepared for the OSCEs	7%	39%	54%
	Watching student OSCE video examples helped me prepare for the OSCE	5%	9%	86%
	Filming, watching and reflecting on my role play video allowed me to learn and improve on my skills for the OSCE	4%	4%	92%
	Reviewing and providing feedback on my peers’ role play videos allowed me to learn and improve on my skills for the OSCE	9%	4%	87%

**Table 2:** Multiple regression of OSCE teaching activities (independent variables) and OSCE performance (dependent variables).

Independent Variables	Overall OSCE Mark		OSCE Communication Mark		OSCE Analytical Checklist Mark	
	Std. B	*p*	Std. B	*p*	Std. B	*p*
Summative pre-workshop video and reflection submission	0.284	0.001	0.306	0.001	0.239	0.007
Workshop attendance	0.088	0.294	0.096	0.253	0.048	0.566
Summative post-workshop MCQ quiz mark	−0.011	0.894	−0.049	0.573	0.060	0.488

## Data Availability

The original data are stored by the authors. Participants did not consent to have their raw data made publicly available. However, data on this study may be available upon reasonable request in various forms.
